# Effect of anaesthesia on the perioperative outcomes of pelvi-acetabular fracture surgeries in the apex trauma centre of a developing country–a retrospective analysis

**DOI:** 10.1186/s41038-015-0011-y

**Published:** 2015-07-31

**Authors:** Naveen Yadav, Suma Rabab Ahmad, Nisha Saini, Babita Gupta, Chhavi Sawhney, Rakesh Garg, Vijay Sharma, Vivek Trikha

**Affiliations:** 1 Department of Anaesthesia, Jai Prakash Narayan Apex Trauma Centre, All India Institute of Medical Sciences, New Delhi, India; 2 Department of Anaesthesia, Pain and Palliative Medicine, Dr. B.R. Ambedkar Institute Rotary Cancer Hospital, All India Institute of Medical Sciences, New Delhi, India; 3 Department of Orthopaedics, Jai Prakash Narayan Apex Trauma Centre, All India Institute of Medical Sciences, New Delhi, India

**Keywords:** Anaesthesia, Pelvi-acetabular, Fracture, Perioperative, Outcomes

## Abstract

**Background:**

Regional anaesthesia has been proposed to reduce intraoperative blood loss, duration of hospital stay and in-hospital complications with improved postoperative pain control. General anaesthesia is advantageous for prolonged surgeries. We hypothesized that combined regional and general anaesthesia would offer advantages of both in pelvi-acetabular fracture surgeries.

**Methods:**

We identified 71 patients who underwent open reduction and internal fixation of pelvi-acetabular fractures from May 2012 to 2013 in our trauma centre. We excluded patients with incomplete records (*n* = 4) and other injuries operated along (*n* = 8). Hence, 59 patients were divided into three groups: G group (general anaesthesia), R group (regional anaesthesia) and GR group (combined regional and general anaesthesia).

Main outcome measurements studied were intraoperative blood loss, duration of hospital stay, duration of surgery and intraoperative and postoperative complications.

**Results:**

No differences were obtained in between the groups in terms of age, gender, Injury Severity Score, number of comorbidities, or duration from injury to surgery. No significant differences were found between the three groups for intraoperative blood loss, days of hospital stay and duration of surgery. Intraoperative and postoperative complications were also comparable between the groups (*p* > 0.05).

**Conclusions:**

There is no specific significant advantage of the technique of anaesthesia on the observed perioperative complications in pelvi-acetabular fracture surgeries.

## Background

Pelvi-acetabular fractures (PAF) represent around 3 % of orthopaedic injuries [1]. However, these fractures are associated with an increased risk of morbidity and mortality among trauma patients [2]. Overall mortality from pelvi-acetabular fractures ranges from 5 to 16 %. The mortality rate for acetabular fractures is 3 % [3]; while open pelvic fractures are associated with a mortality rate of up to 45 % [1, 4]. Elderly patients aged greater than 65 with pelvic fractures have a mortality rate of approximately 20 % [5]. Early definitive fixation of unstable pelvis and acetabular fractures in multiply injured patients reduces morbidity [6].

Previous studies have theorized a potential benefit of regional anaesthesia (RA) over general anaesthesia (GA) in orthopaedic surgeries [7]. Combined general and regional anaesthesia (GRA) may be proposed to offer the advantages of both; however, this fact has not been established [8]. As far as we know, no previous study has conclusively demonstrated the difference in perioperative outcomes of GA, RA and GRA in PAF surgeries. So, we conducted the retrospective analysis of the patients who were operated for pelvi-acetabular fractures with an aim to find the difference in outcome with regards to technique of anaesthesia.

## Methods

### Study design

The analysis was conducted after obtaining institutional ethics clearance. We used a retrospective exploratory study design. Our patients comprised of adults between age 18 and 65 years admitted to level 1 trauma centre. Data records of all patients operated in our centre for pelvi-acetabular fracture were analysed, and information relevant to present study was noted. At our centre, such fractures are variably operated under different anaesthetic technique including general anaesthesia, regional anaesthesia (combined spinal and epidural block), or combined regional (epidural anaesthesia) and general anaesthesia based on the discretion of the attending anaesthesiologist.

### Data collection

All patients operated in our centre from May 2012 to May 2013 were analysed for the study purpose. The database included the medical records including the medical case registers, patients’ hospital file and the coding history entered in a preformed performa. The demographic parameters like age, sex, mechanism of injury, time of injury and type of fracture were noted. The other parameters which were noted included type of anaesthetic technique, mortality, duration of hospital stay, cardiovascular morbidity (hypotension with mean arterial pressure <60 mmHg, significant arrhythmias and myocardial infarction), incidence of deep vein thrombosis, pulmonary embolism, intraoperative blood loss, intra- and postoperative blood requirement, postoperative analgesic requirement, duration of surgery and adverse effects (postoperative nausea vomiting, pruritus, sedation, urinary retention and respiratory depression). Rigorous data validation checks were performed to ensure accuracy of data entries. The patients with incomplete records or who had associated other injuries were excluded from the study. Injury Severity Score (ISS) was calculated and noted. To calculate an ISS, the body was divided into six body regions which were head or neck, face, chest, abdomen or pelvic contents, extremities or pelvic girdle and external. Each body region is given a severity score from 1 to 6. To calculate an ISS, the highest severity code in each of the three most severely injured ISS body regions was squared and added. (ISS = *A*^2^ + *B*^2^ + *C*^2^ where *A*, *B* and *C* were the highest scores of the three most injured ISS body regions).

### Study sample

The patients who underwent pelvi-acetabular fracture surgeries in the form of open reduction and internal fixation of posterior and anterior column and total hip replacement under general or regional or combined regional and general anaesthesia were segregated into three groups: G group (general anaesthesia), R group (regional anaesthesia) and GR group (combined regional and general anaesthesia).

### Statistical analysis

Data was analysed by STATA 12. The data was represented in mean ± SD or median (frequency, percentage). Chi-square or Fisher’s exact test was applied to compare the qualitative variable among the group. ANOVA or Kruskal-Wallis test was used to compare the quantitative variable among the group. *p* value <0.05 was considered statistically significant. The percentage of each mechanism of injury, comorbidity or intraoperative and postoperative complication in every group was calculated, and then Fisher’s exact test was applied for comparison.

## Results

A total of 71 patients who underwent open reduction and internal fixation of pelvi-acetabular fractures over 1 year from May 2012 to May 2013 in our trauma centre were identified. We excluded patients who had incomplete records (*n* = 4) and those with other injuries operated along (*n* = 8). Hence, 59 patients were segregated into three groups: G group (general anaesthesia), R group (regional anaesthesia) and GR group (combined regional and general anaesthesia).

Demographic data was analysed. No statistically significant differences were obtained in between the groups in terms of age, gender, Injury Severity Score, mechanism of injury, type of fracture, associated injuries or duration from injury to surgery (Table 1) (*p* > 0.05). Comorbidities were also comparable between the groups (Table 2) (*p* > 0.05).

**Table 1:** Patient demographic data (*N* = 59)

	GR group (*n* = 14)	R group (*n* = 30)	G group (*n* = 15)	*p* value
Mean age ± SD (years)	30.64 ± 11.86	38.70 ± 14.96	32.27 ± 10.15	0.114^b^
Percent males (*n*)	71.4 % (10)	93.3 % (28)	93.3 % (14)	0.106^c^
Mean ISS ± SD	20.14 ± 7.37	20.83 ± 12.77	26.00 ± 10.56	0.276^b^
Mechanism of injury (no.)				0.593^c^
• Fall	4	11	3	
• RTA^a^	8	16	11	
• Railway Tract Injury	0	1	1	
• Pedestrian	2	2	0	
Fracture type				0.308^c^
• Pelvic	10	21	12	
• Acetabular	1	6	0	
• Combined Pelvic Acetabular	3	3	3	
Percent of associated injury				>0.05^c^
• Femur fracture	6.6	8.1	6.7	
• Sciatic nerve injury	0	3.3	6.7	
• Sacral fracture	7.1	6.7	0	
• Bladder rupture	0	6.7	0	
• Organ injury	2.1	1.1	0	
• Posterior dislocation of the hip	21.4	10	20	
• Degloving injury	0	3.3	0	
Time from injury to surgery (days)				0.242^c^
• <1	1	0	1	
• 1 to 15	11	23	9	
• 15 to 30	1	2	4	
• >30	1	5	1	

^a^ *RTA* Road Traffic Accident

^b^ANOVA/Kruskal-Wallis test

^c^Fisher’s test/chi-square test

**Table 2:** Incidence of comorbidities

	GR group (*n* = 14)	R group (*n* = 30)	G group (*n* = 15)	*p* value
Diabetes mellitus (DM)	0	1	1	
Hypertension (HT)	1	2	2	
Both (DM and HT)	1	1	0	
Obesity/OSA	1	0	0	
Pleural effusion/consolidation	1	2	0	
COPD/bronchiectasis/asthma	1	2	0	
ECG/cardiac changes	1	0	2	
Total	6	8	5	>0.05^a^

*OSA* obstructive sleep apnea, *COPD* chronic obstructive pulmonary disease

^a^Fisher’s Test

**Fig. 1:**
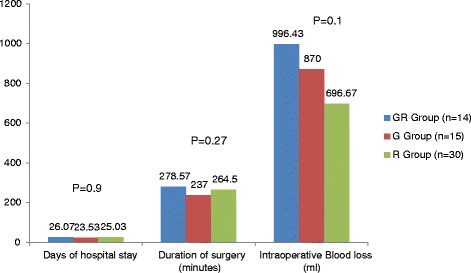
Comparison of perioperative outcomes (mean ± SD)

The perioperative outcomes were also comparable (Fig. 1) (*p* > 0.05). No statistically significant differences were found between the groups for days of hospital stay (GR, 26.07 ± 18.92; R, 25.03 ± 15.87; G, 23.53 ± 9.80; *p* = 0.90) and duration of surgery (GR, 278.57 ± 57.12 min; R, 264.5 ± 79.53 min; G, 237 ± 63.35 min; *p* = 0.27). No statistically significant differences were found between the groups for intraoperative blood loss (GR, 996.43 ± 549.29 ml; R, 696.67 ± 500.33 ml; G, 870 ± 516.79 ml; *p* = 0.18). However, numerically, patients in the R group had the least blood loss as compared to the GR and G groups.

Intraoperative crystalloid, colloid and packed red blood cells (PRBC) requirement were comparable indicating stable hemodynamic intraoperatively (*p* > 0.05) (Table 3). Intraoperative complications were also comparable between the groups (*p* > 0.05). However, patient position approached statistical significance among the three groups (*p* = 0.002). Most of the surgeries done in a supine position were done under regional anaesthesia. However, this difference did not affect the perioperative outcome.

**Table 3:** Comparison of intraoperative complications, crystalloid & colloid requirement, patient position

	GR group (*n* = 14)	R group (*n* = 30)	G group (*n* = 15)	*p* value
Intraoperative complications				>0.05^a^
• Bronchospasm	0	0	1	
• Hypotension	1	1	0	
• Arrhythmia	0	1	1	
Mean intraoperative crystalloid ± SD (ml)	3128.57 ± 1067.30	2563.33 ± 862.83	2966.67 ± 833.81	0.121^b^
Mean intraoperative no. of 500 ml colloid (ml) ± SD	1.50 (750) ± 0.52	1.70 (833.33) ± 0.53	1.60 (833.33) ± 0.63	0.532^b^
Mean intraoperative PRBC transfused ± S.D	1.43 ± 1.09	1.03 ± 1.1	1.07 ± 1.03	0.511^b^
Position				0.002^a^
• Supine	3	16	4	
• Prone	6	10	4	
• Lateral	5	4	7	

^a^Fisher’s test/chi-square test

^b^ANOVA/Kruskal-Wallis test

Postoperative complications and patients requiring intensive care unit admission were also comparable between the groups (*p* > 0.05) (Table 4).

**Table 4:** Incidence of postoperative complications and ICU admission

	GR group (*n* = 14)	R group (*n* = 30)	G group (*n* = 15)	*p* value
Myocardial ischaemia, Arrhythmia	2	1	0	
Pneumonia and other infection	5	3	2	
Bleeding	5	4	3	
Hypotension	2	1	0	
Deep venous thrombosis	0	0	1	
Pulmonary embolism	0	0	0	
Resurgery	0	3	1	
ICU admission	0	1	3	
Total	14	13	10	>0.05^a^

^a^Fisher’s test/chi-square test

## Discussion

We observed from our study that the technique of anaesthesia including regional anaesthesia, general anaesthesia or a combination of both did not have impact on major outcome after the surgical intervention for pelvi-acetabular fractures presenting to our apex trauma centre.

Perioperative management during pelvi-acetabular fracture surgeries determine the outcomes in terms of morbidity, mortality and eventually in-hospital duration of stay and treatment cost. There is an increase in the risk of complications and life-threatening events after a major surgery like pelvi-acetabular fracture surgeries. Conflict prevails whether the type of anaesthesia has any substantive effect on these risks [9, 10].

Neuraxial anaesthesia has several physiological effects like sympatholysis, and hence, we expect it to improve outcome by decreasing the blood loss and improved perioperative analgesia [11–13]. The multifactorial mechanisms for the beneficial effects of regional anaesthesia includes altered coagulation [14], increased blood flow, improved ability to breathe free of pain and reduction in surgical stress responses [15]. In particular, neuraxial blockade but not general anaesthesia substantially reduces “stress response” of major surgery [11, 16]. Some studies have demonstrated the benefit of regional anaesthesia versus general anaesthesia with regard to perioperative complications which notably reduced operating time, lesser blood loss and major postoperative complications [7]. However, pertaining to pelvi-acetabular fracture surgeries, as to our knowledge, supportive data is lacking.

Previous studies have shown that improved survival in patients randomized to neuraxial blockade [7, 17, 18]. In the regional group, they also found reductions in risk of transfusion requirement, venous thromboembolism [19], myocardial infarction, bleeding complications, pneumonia, respiratory depression and renal failure. They suggested that the benefits are principally due to the use of neuraxial blockade rather than avoidance of general anaesthesia. However, there was uncertainty of a clear evidence of these effects, in terms of the type of surgical group or the type of neuraxial blockade. Some of the previous studies have analysed that neuraxial block definitely reduces surgical blood loss considering certain surgeries [20]. Although a review has negated this beneficial effect of regional anaesthesia with regard to total knee arthroplasty [21], others have concluded that this effect do not usually lead to a reduction in the number of transfused patients except for patients undergoing total hip replacement and spinal fusion [22]. Hence a conflict prevails.

In the present analysis, the comparison of perioperative outcomes was similar in all the three groups. The days of hospital stays were equivalent so as the duration surgery in all the three groups. The intraoperative blood loss was comparable in all the three groups, and the difference was not significant. The least blood loss was seen in regional anaesthesia group, and this could be attributed to sympatholysis causing hypotension which subsequently would have caused decreased blood loss. With no change in hospital stay and equivalent operation time, it can be inferred that the type of anaesthesia may not affect the outcome of these pelvi-acetabular fractures.

The intraoperative complication like bronchospasm, hypotension and arrhythmia was similar in all the groups. This could be attributed to young demographic profile of the patients taken in our study [23]. The increase in average age in all the groups could have some significant cardiovascular changes in the general anaesthesia group and that could have been statistically significant [24]. The crystalloid and colloid requirement in all the groups is comparable. This could probably be because the regional anaesthesia does not cause marked hypotension as predicted, and therefore, the requirement of bolus crystalloid and colloid was not needed in our analysis. However, patient position approached statistical significance among the three groups (*p* = 0.002). Most of the surgeries done in a supine position were done under regional anaesthesia, as patients whose fractures were difficult to approach in a supine position were approached by a prone position and were give general anaesthesia or general anaesthesia and regional anaesthesia. However, this difference in position did not affect the perioperative outcome in all the three groups.

Postoperative complications and intensive care unit (ICU) admissions were comparable in all the three patient groups. Although, in G group, three patients required ICU admission, one patient had deep vein thrombosis and one had resurgery. Thus, it can be said that regional anaesthesia has no clear cut advantage in reducing the postoperative complication and ICU stay complying with Rashid et al. in hip fracture surgery [25].

The strength our study is that the scarce research is present in comparison of regional anaesthesia, general anaesthesia and combined regional general anaesthesia in pelvi-acetabular fracture surgeries. We compared not only the intraoperative complication but also the postoperative complication, ICU stay and total length of hospital stay. Our study may be limited by the fact that it was not a randomized prospective trial, and thus, bias may be present in view of retrospective analysis. Moreover our study did not include postoperative analgesia because of insufficient data. In addition, in our study, most of the patients were younger age group and may not be applicable to elderly patients. However, such traumatic injuries are more commonly seen in younger population in Indian subcontinent.

## Conclusions

Therefore, we conclude that the regional anaesthesia alone or with general anaesthesia may have no impact in reducing intraoperative blood loss, cardiovascular complications intraoperatively and postoperatively, intraoperative crystalloid and colloid requirement, PRBC requirement during surgery, duration of hospital stay and ICU stay compared to patient given general anaesthesia alone in pelvi-acetabular fracture surgeries.

## Abbreviations

GA: general anaesthesia
GRA: combined general and regional anaesthesia
ICU: intensive care unit
ISS: Injury Severity Score
PAF: pelvi-acetabular fracture
PRBC: packed red blood cells
RA: regional anaesthesia

## References

[1] Grotz MR, Allami MK, Harwood P, Pape HC, Krettek C, Giannoudis PV (2005). Open pelvic fractures: epidemiology, current concepts of management and outcome. Injury.

[2] Schulman JE, O’Toole RV, Castillo RC, Manson T, Sciadini MF, Whitney A (2010). Pelvic ring fractures are an independent risk factor for death after blunt trauma. J Trauma.

[3] Giannoudis PV, Grotz MR, Papakostidis C, Dinopoulos H (2005). Operative treatment of displaced fractures of the acetabulum. A meta-analysis. J Bone Joint Surg Br.

[4] Cannada LK, Taylor RM, Reddix R, Mullis B, Moghadamian E, Erickson M (2013). The Jones-Powell Classification of open pelvic fractures: a multicenter study evaluating mortality rates. J Trauma Acute Care Surg.

[5] Dechert TA, Duane TM, Frykberg BP, Aboutanos MB, Malhotra AK, Ivatury RR (2009). Elderly patients with pelvic fracture: interventions and outcomes. Am Surg.

[6] Vallier HA, Cureton BA, Ekstein C, Oldenburg FP, Wilber JH (2010). Early definitive stabilization of unstable pelvis and acetabulum fractures reduces morbidity. J Trauma.

[7] Rodgers A, Walker N, Schug S, McKee A, Kehlet H, van Zundert A (2000). Reduction of postoperative mortality and morbidity with epidural or spinal anaesthesia: results from overview of randomised trials. BMJ.

[8] Kulkarni S (2002). Anaesthesia for surgical management of fractures of acetabulum. Indian J Orthop.

[9] Parker MJ, Handol HH, Griffiths R (2001). Anaesthesia for hip fracture surgery in adults. Cochrane Database Syst Rev.

[10] Luger TJ, Kammerlander C, Gosch M, Kammerlander-Knauer U, Roth T, Kreutziger J. Neuroaxial versus general anaesthesia in geriatric patients for hip fracture surgery: does it matter? Osteoporos Int. 2010;21:S555–72. 10.1007/s00198-010-1399-7 21057995

[11] Kehlat H, Cousins M, Bridenbaugh P (1988). Modification of responses to surgery by neural blockade: clinical implications. Neural blockade in clinical anaesthesia and management of pain.

[12] Fleming I, Egeler C (2014). Regional anaesthesia for trauma: an update. Contin Educ Anaesth Crit Care Pain.

[13] Egol KA, Soojian MG, Walsh M, Katz J, Rosenberg AD, Paksima N (2012). Regional anesthesia improves outcome after distal radius fracture fixation over general anesthesia. J Orthop Trauma.

[14] Kettner SC, Willschke H, Marhofer P (2011). Does regional anaesthesia really improve outcome?. Br J Anaesth.

[15] Reinhart K, Foehring U, Kersting T, Schaefer M, Bredle D, Hirner A, et al. Effects of thoracic epidural anesthesia on systemic hemodynamic function and systemic oxygen supply–demand relationship. Anesth Analg. 1989;69:360–9. 2774232

[16] Nimmo SM (2004). Benefit and outcome after epidural analgesia. Contin Educ Anaesth Crit Care Pain.

[17] Wijeysundera DN, Beattie WS, Austin PC, Hux JE, Laupacis A (2008). Epidural anaesthesia and survival after intermediate-to-high risk non-cardiac surgery: a population-based cohort study. Lancet.

[18] Hu S, Zhang ZY, Hua YQ, Li J, Cai ZD (2009). A comparison of regional and general anaesthesia for total replacement of the hip or knee: a meta-analysis. Bone Joint Surg [Br].

[19] Urwin SC, Parker MJ, Griffiths R (2000). General versus regional anaesthesia for hip fracture surgery: a meta-analysis of randomized trials. Br J Anaesth.

[20] Attari MA, Mirhosseini SA, Honarmand A, Safavi MR (2011). Spinal anesthesia versus general anesthesia for elective lumbar spine surgery: a randomized clinical trial. J Res Med Sci.

[21] Macfarlane AJR, Prasad GA, Chan VWS, Brull R (2009). Does regional anesthesia improve outcome after total knee arthroplasty?. Clin Orthop Relat Res.

[22] Guay J (2006). The effect of neuraxial blocks on surgical blood loss and blood transfusion requirements: a meta-analysis. JClinAnesth.

[23] Turrentine FE, Wang H, Simpson VB, Jones RS (2006). Surgical risk factors, morbidity, and mortality in elderly patients. J Am CollSurg.

[24] Braz L, Braz DG, Cruz DS, Fernandes LA, Módolo NS, Braz JR (2009). Mortality in anesthesia: a systematic review. Clinics (Sao Paulo).

[25] Rashid RH, Shah AA, Shakoor A, Noordin S (2013). Hip fracture surgery: does type of anesthesia matter?. Biomed Res Int.

